# Zenker's diverticulum: a case report and literature review

**DOI:** 10.11604/pamj.2014.17.267.4173

**Published:** 2014-04-12

**Authors:** Moawia Elbalal, Abu Baker Mohamed, Anas Hamdoun, Khalid Yassin, Elhadi Miskeen, Osman Khalaf Alla

**Affiliations:** 1 Gezira Centre for GIT endoscopy & laparoscopic surgery, Wad Medani, Sudan; 2 National cancer Institute (NCI), Gezira, Sudan; 3 Alneelain University Faculty of Medicine, Sudan; 4 University of Gezira Faculty of Medicine

**Keywords:** Oropharyngeal dysphagia, pharyngeal pouch, Zenker′s diverticulum

## Abstract

The pharyngeal pouch (Zenker's diverticulum) is a diverticulum of the mucosa of the pharynx, just above the cricopharyngeal muscle (i.e. above the upper sphincter of the oesophagus). It occurs commonly in elderly patients (over 70 year) and the typical symptoms include dysphagia, regurgitation, chronic cough, aspiration and weight loss. We are reporting a case of an oropharyngeal dysphagia due to a Zenker's diverticulum in 75 years old Sudanese man with a chronic history of dysphagia for solids. The pathophysiology of Zenker's diverticulum, clinical presentation, and management are reviewed.

## Introduction

The oesophagus is a muscular tube 25 cm long which extends from cricoid cartilage to the cardiac orifice of the stomach. It joins the larynx to the stomach. It has an upper and lower sphincter. Peristaltic wave propels the food bolus into the stomach. [1] Current knowledge of the swallowing mechanism is derived mainly from radiographic studies, which has been used since early 1900s. Plain films of the pharynx were replaced in 1930s by cineradiography which was subsequently replaced by video fluoroscopy in 1970s. Deglutition can be divided into four phases: [1] oral preparatory phase [2] oral voluntary phase [3] pharyngeal phase and [4] oesophageal phase. The pharyngeal swallowing response is rapid, highly coordinated activity that results in velopharyngeal closure, laryngeal elevation and closure, opening of the upper oesophageal sphincter (UOS), tongue loading, tongue pulsion, and pharyngeal clearance. [2]

Oropharyngeal dysphagia results from either oropharyngeal swallowing dysfunction or perceived difficulty in the process of swallowing. One of those causes is pharyngeal pouches (Zenker's diverticulum) that occur most commonly in elderly patients (over 70years) and typical symptoms include dysphagia, regurgitation, chronic cough, aspiration and weight loss. The etiology remains unknown but theories center upon a structural or physiological abnormality of the cricopharynx. The diagnosis is easily established by barium studies. Treatment is surgical via endoscopic or external cervical approach and should include cricopharyngeal myotomy. Unfortunately phyaryngeal pouch has long been associated with significant morbidity; partly due to the surgery itself and the fact that the majority of patients are elderly and often have general medical problems. External approaches are associated with high complication rates than endoscopic procedures. Recently treatment by endoscopic stapling & diverticulotomy has been increasingly popular as it has distinct advantages, although long term results are not yet available. The small risk of developing carcinoma within a pouch that is not excised remains a continuous issue and an argument for long term follow up or treating the condition by external excision, particularly in younger patients. [3]

## Patient and observation

75 years-old Sudanese male presented with dysphagia which started for solid food over one year. It was of gradual onset, progressive but sometimes food passes with difficulty, later he started to have difficulty in swallowing fluids. No difficulty in initiating swallowing, but he sometimes feels as if food sticks up in his neck. He had no odynophagia, but he noticed some weight loss. He gave no history of dysphonia, nasal regurgitation or dysarthria. His musculoskeletal system is free of arthritis or skin rash. He had no similar condition in his family members. During examination patient was ill, wasted, slightly pale, but not jaundiced or cyanosed. His chest, cardiovascular, abdomen and CNS were all normal.

**Figure 1:**
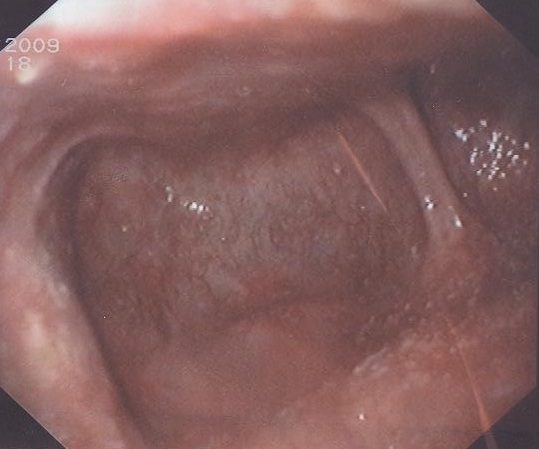
1O GD showing 2 diverticula (arrows)

**Figure 2:**
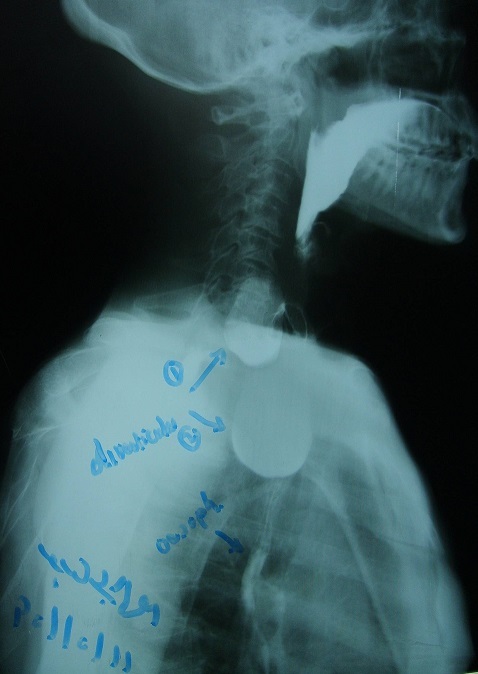
Barium swallow showing 2 oesophageal diverticula

**Figure 3:**
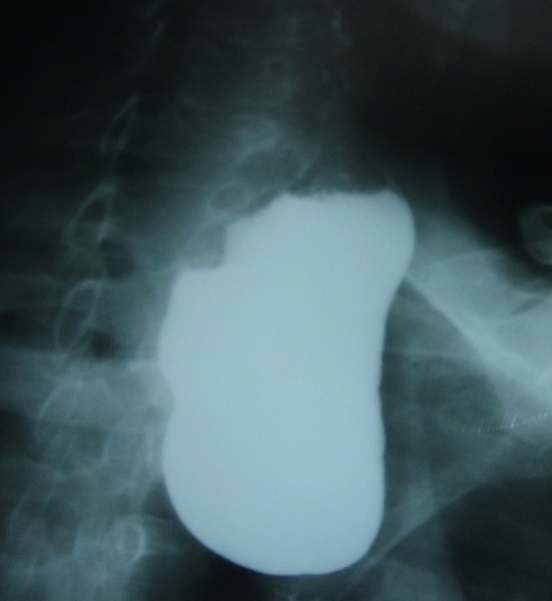
Barium swallow showing the large diverticulum

From the above mentioned clinical scenario, the suspicion of oropharyngeal dysphagia has arisen. An upper gastrointestinal endoscopy was performed, which revealed two oesophageal diverticula just at 10 cm from the oral cavity. Both of them showed a smooth mucosa in their inner aspect. The oesophageal lumen was situated very laterally with a great difficulty to pass the scope through it (Figure 1). Barium swallow was requested, which revealed two oesophageal diverticula: One was small and laterally situated and sitting against the fifth cervical vertebra in keeping with Zenker's diverticulum, and the other is rather large sitting below it. Small amount of Barium could be seen passing through the rest of the oesophagus distal to the two diverticula (Figure 2, Figure 3). Later on, the patient was built up by i.v fluids, which led to improvement of his general condition, then he was referred for a surgical opinion. Unfortunately, the patient disappeared before any surgical intervention.

## Discussion

Oropharyngeal dysphagia has high morbidity, mortality & cost. Although epidemiological data are scanty, estimates of the prevalence of dysphagia among individuals older than 50 years range from 16% to 22% [4, 5]. The consequences of oropharyngeal dysphagia can be severe: dehydration, malnutrition, aspiration, choking, pneumonia and death. A rare cause of this condition is Zenker's diverticulum which shows an annual incidence of 2 per 100,000 per year in UK [3]. It is defined as an out-pouching of the mucosa through Killian's triangle, an area of muscular weakness between the transverse fibers of the cricopharyngeus & the oblique fibers of the lower inferior constrictor. It was first described by Ludlow in 1767 [6]. Zenker & Van Ziemssen subsequently reviewed the world literature in 1877, and since then this kind of diverticulum has been called Zenker's diverticulum [7].

Zenker's diverticulum (ZD) is caused by motor abnormalities of the oesophagus including spasm, achalasia, lower oesophageal sphincter hypertension, or nonspecific abnormalities [8, 9]. The most appropriate hypothesis is the high intrabolus pressure & the resistance to swallowing due to abnormalities of the upper oesophageal sphincter (UOS). Zenker's diverticulum (ZD) is usually discovered in older adults, although they have been described in children. Most patients present after the age of 60 (often above age 75), having had symptoms ranging from weeks to years. For unclear reason, the majority of patients are males [10]. Typical symptoms of Zenker's diverticulum (ZD) include: dysphagia, regurgitation, chronic cough, aspiration & weight loss [3]. Zenker's diverticulum (ZD) is usually diagnosed with barium examination. Like our case a second diverticulum is present in approximately 1 to 2% of patients but is usually much smaller than the first [10]. In contradistinction to this observation our old man had a large second diverticulum.

Zenker's diverticulum (ZD) can produce a variety of symptoms & complications such as aspiration pneumonia. In addition, a very rare complication is the occurrence of the carcinoma in the diverticulum [11]. Ulceration & bleeding due to retained aspirin has been described [12]. Caution must be used during endoscopy or passage of nasogastric tubes because of the risk inadvertent perforation of the diverticulum. The mainstay of treatment of symptomatic Zenker's diverticulum (ZD) has been surgery [13, 14]. However, in Europe, nonsurgical minimally invasive methods have been increasingly used. The operation is either one or two stage type including cricopharyngeal myotomy & diverticulectomy. Recently, treatment by endoscopic stapling diverticulotomy has been increasingly popular as it has distinct advantages [3].

## Conclusion

Whenever faced by a case of an oropharyngeal dysphagia, physicians should bear in their minds the possibility of encountering Zenker's diverticulum. Barium swallow should be the first modality of investigation. The patient should be rotated during the examination to delineate small diverticula. Careful endoscopy should be carried out in confirming the diagnosis.
